# Physiologically relevant culture medium Plasmax improves human placental trophoblast stem cell function

**DOI:** 10.1152/ajpcell.00581.2022

**Published:** 2023-03-06

**Authors:** Giulia Avellino, Ruhi Deshmukh, Stephanie N. Rogers, D. Stephen Charnock-Jones, Gordon C. S. Smith, Saverio Tardito, Irving L. M. H. Aye

**Affiliations:** ^1^Department of Obstetrics and Gynaecology, University of Cambridge, NIHR Cambridge Comprehensive Biomedical Research Centre, Cambridge, United Kingdom; ^2^Department of Physiology, Development and Neuroscience, Centre for Trophoblast Research (CTR), University of Cambridge, Cambridge, United Kingdom; ^3^Cancer Research UK Beatson Institute, Garscube Estate, Glasgow, United Kingdom; ^4^School of Cancer Sciences, University of Glasgow, Glasgow, United Kingdom

**Keywords:** culture medium, placenta, stem cells

## Abstract

Human trophoblast cultures provide powerful tools to model key processes of placental development. In vitro trophoblast studies to date have relied on commercial media that contains nonphysiological levels of nutrients, and the impact of these conditions on trophoblast metabolism and function is unknown. Here, we show that the physiological medium (Plasmax) with nutrient and metabolite concentrations recapitulating human plasma improves human trophoblast stem cell (hTSC) proliferation and differentiation compared with standard medium (DMEM-F12). hTSCs cultured in Plasmax-based medium also show altered glycolytic and mitochondrial metabolism, as well as reduced S-adenosylmethionine/S-adenosyl-homocysteine ratio compared with DMEM-F12-based medium. These findings demonstrate the importance of the nutritional environment for phenotyping cultured human trophoblasts.

## Graphical abstract

**Figure:**
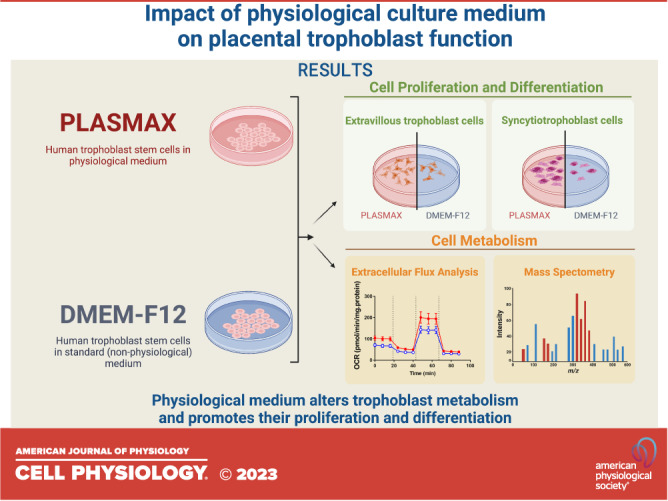

## INTRODUCTION

Major pregnancy complications originate from poor placental development in the first trimester of pregnancy (1). During this period, placental trophoblasts undergo rapid and extensive transformation. This process depends on the proper coordination of trophoblast stem cell proliferation and differentiation into the syncytiotrophoblast (STB) or extravillous trophoblast (EVT) lineages. No animal models replicate the unique structure and development of the human placenta. Therefore, trophoblast cultures provide fundamental tools for mechanistic studies of important process regulating human placental development.

In recent years, there have been significant developments in in vitro human trophoblast studies such as the derivation of human trophoblast stem cells and organoids (2–4). These developments were made possible by the identification of key growth factors, supplements, and matrices that support the culture of these novel in vitro models. Despite these advances, researchers continue to rely on culture media that were not designed to reflect the physiological environment of human cells. Mammalian culture media were originally formulated for the continued culture of cells with minimal amounts of nutrients and serum, e.g., Eagle’s minimal essential medium (5). Modifications to this original formulation, such as Dulbecco’s modified Eagle’s minimal essential medium (DMEM), increased the concentrations of selected nutrients up to fourfold to prevent nutrient depletion, allowing for cultures to remain unattended over extended periods (6). Another commonly used medium Ham’s F12, was designed for the growth of Chinese Hamster Ovaries (7). Even though the DMEM-F12 medium poorly resembles the physiological environment, most trophoblast studies (>70%) are performed using this medium (8). One important question is whether these deviations in nutrients and electrolytes from physiological levels can impact trophoblast behavior.

Recently, more complex culture media were developed, using nutrients, metabolites, and other organic components at concentrations found in human plasma. These media called Plasmax (9) and human plasma-like medium (10) were thus designed to resemble the in vivo human physiological metabolic environment. Previous studies comparing cells cultured with physiological media versus standard media (e.g., DMEM-F12) revealed profound differences in metabolism (9–11). Although these media have been mainly employed for studies with cancer cells, which characteristically exhibit altered metabolism, a recent report have demonstrated that physiological media support the proliferation of primary mesenchymal stromal cells (12).

In this study, we compared the effects of the physiological medium (Plasmax) with the commonly used DMEM-F12 on human trophoblast stem cells (hTSCs). We determined whether Plasmax altered the proliferation and differentiation capacity of hTSCs and profiled their metabolism under this more physiological culture condition.

## METHODS

hTSCs were derived from human first-trimester placentas by Okae et al. (2) and lines CT29 and CT30 used in this study were a kind gift from his laboratory. They were cultured in conditions previously described (2) with minor modifications. hTSCs were maintained in their stem-state by culturing in hTSC medium, which consists of 0.3% bovine serum albumin (BSA), 1% insulin-transferrin-selenium-ethanolamine (ITS-X), 0.1 mM 2-mercaptoethanol, 0.2% fetal bovine serum (FBS), 1.5 µg/mL ascorbic acid, 100 µg/mL Primocin (antibiotic/antimycotic), 0.8 mM valproic acid, 50 ng/mL epidermal growth factor, 5 µM Y27632 (ROCK inhibitor), 2 µM CHIR99021 (GSK3 inhibitor), 1 µM SB43154 (TGF-β inhibitor), and 0.5 µM A83-01 (ALK inhibitor). hTSCs were plated at 1 × 10^5^ cells in a six-well dish precoated with 5 µg/mL collagen IV.

STB differentiation was carried out by plating 1 × 10^5^ hTSCs in a six-well dish precoated with 2.5 µg/mL collagen IV, in STB medium which consists of 0.1 mM 2-mercaptoethanol, 0.3% BSA, 1% ITS-X supplement, 100 µg/mL Primocin, 2.5 µM Y27632, 2 µM forskolin (cAMP agonist), and 10% FBS.

For EVT differentiation, 7.5 × 10^4^ hTSCs were seeded in a six-well dish precoated with 1 µg/mL of collagen IV, in EVT medium which consists of 0.1 mM 2-mercaptoethanol, 0.3% BSA, 1% ITS-X supplement, 4% knockout serum replacement, 100 µg/mL Primocin, 2.5 µM Y27632, 0.5 µM A83-01, and 100 ng/mL Neureglin-1. Matrigel was then added to a final concentration of 2%. On *day 3*, the medium was replaced with the same EVT medium without Neureglin-1, and Matrigel was added to a final concentration of 0.5%. On *day 6*, the medium was replaced with EVT medium omitting Neureglin-1 and knockout serum replacement, and Matrigel was added to a final concentration of 0.5%.

All cells (i.e., hTSCs, STB, and EVTs) were grown in either standard medium (DMEM-F12-based) or physiological medium (Plasmax-based) containing the supplements described earlier, and the medium was replaced daily.

ITS-X (#51500-056), 2-mercaptoethanol (#31350), knockout serum replacement (#10828010), and DMEM-F12 (#11320) were from Thermo Fisher Scientific. BSA (#A8412), valproic acid (#P4543), FBS (#F7524), l-ascorbic acid (#A8960), and forskolin (#93049) were from Sigma Aldrich. Y-27632 (#Y0503), SB43154 (#1614), and A83-01 (#SML0788) were from Stem Cell Technologies. Primocin (#ant-pm-1) was acquired from Invivogen, EGF (#AF-100-15) from Peprotech, Neureglin-1 (#5218SC) from Cell Signaling Technology, Matrigel (#356231) from Corning, and Plasmax (#156371) from Cancer tools.

### Cell Proliferation Assays

hTSCs were initially cultured in DMEM-F12-based medium for maintenance. For proliferation assays, hTSCs were trypsinized and replated at 0.1 million cells in either physiological (Plasmax-based) or standard (DMEM-F12-based) medium. The concentration of growth factors and supplements used for hTSC maintenance (described earlier) remained the same for both media. Cells were then trypsinized and counted after 1–4 days in culture. Experiments were repeated in four independent replicates (i.e., cell passages).

### Human Chorionic Gonadotrophin-β ELISA

hTSCs were differentiated into STB as described above and conditioned media was collected after 1, 3 and 6 days of culture in DMEM-F12-based medium or Plasmax-based medium. Human chorionic gonadotrophin (hCG) secretion was measured using the hCG-β DuoSet ELISA from R&D Systems (#DY9034) according to manufacturer’s instructions. hCG-β concentrations were normalized to cellular protein concentrations in each well. Experiments were repeated in four independent replicates (i.e., cell passages).

### Analysis of Syncytial Fusion by Immunofluorescence Microscopy

Fusion of hTSCs into a syncytium during STB differentiation was assessed by quantification of the distribution of E-cadherin and nuclei in cells after fixation and immunostaining as previously described with modifications (13). hTSCs were plated in eight-well chamber slides (ibidi, #80806) at 7.5 × 10^3^ cells per well. Following STB differentiation, cells were fixed in 4% paraformaldehyde for 10 min and permeabilized in 0.1% Triton X100. Following washes in PBS-Tween (0.1%), fixed cells were blocked in 10% goat serum for 30 min before incubating with 10 µg/mL of anti-E-cadherin (Life Technologies, #13–1700) overnight. Cells were then washed and incubated in 2 µg/mL Alexa Fluor 488-conjugated anti-rabbit IgG (Life Technologies, #A-21441) for 2 h. Nuclei were stained using the ProLong Glass Antifade Mountant containing DAPI (Life Technologies, #P36983). Cells were imaged using the Nikon Eclipse Ti2 inverted microscope. Analysis was carried out by calculating the percentage of nuclei localized within the syncytium (defined as two or more nuclei within E-cadherin-labeled cell boundary) in four random fields of view per condition (technical replicates). A minimum of 50 nuclei were counted in each field of view. Analysis was carried out by two researchers (G.A and S.R.) who were blinded to the experimental condition. The mean of the four technical replicates was considered to be an independent replicate (i.e., *n* = 1) and each experiment was repeated in five independent replicates (i.e., cell passages) and therefore the total *n* = 5.

### Extracellular Flux Analysis Using Seahorse Bioanalyzer

Mitochondrial activity was determined by measuring the oxygen consumption rate (OCR) using a Seahorse XFe96 bioanalyzer and the Mito Stress Test kit (#103015) according to the manufacturer’s instructions. hTSCs were plated in Seahorse XFe96 microplate (#103729) at 1 × 10^4^ cells/well in hTSC media with DMEM-F12 or Plasmax -based medium and cultured for 3 days with daily media changes. One hour before the start of the assay, the medium was replaced with fresh hTSC media (either DMEM-F12 or Plasmax -based medium). Oligomycin (2 μM), carbonyl cyanide p-trifluoromethoxyphenylhydrazone (FCCP, 2 μM), and rotenone + antimycin (0.5 μM each) were sequentially added according to the experimental protocol (14). Each experimental condition was repeated in eight wells (technical replicates) per plate, and the mean of the replicates considered to be an independent replicate (i.e., *n* = 1). Each experiment was repeated in four independent replicates (i.e., cell passages) and therefore the total *n* = 4. All reagents for extracellular flux analysis were from Agilent Technologies.

### Quantitative PCR Analysis

Total RNA was prepared using RNeasy Plus Mini Kit (#74034, Qiagen). First-strand cDNA was synthesized from total RNA using High-Capacity cDNA Reverse Transcription Kits (#4368814), and quantitative PCR (q-PCR) was performed via Quant-Studio 6 Real-Time PCR system (ThermoFisher Scientific). Q-PCR analysis was performed using Taqman assays for the following genes: *ERVW-1* (#Hs00205893_m1), *CYP19A1* (#Hs00903411_m1), *ERBB2* (#Hs01001580_m1), and *HLA-G* (#Hs00365950_g1). The amount of target mRNA was determined according to the standard curve method and normalized using the geometric mean of *CDC34* (#Hs07288692_g1) and *TBP* (#Hs00427620_m1) as internal controls. Unless otherwise stated, all reagents were from ThermoFisher Scientific.

### Liquid Chromatography Mass Spectrometry

hTSCs were cultured in DMEM-F12 or Plasmax-based medium containing hTSC supplements as described earlier with daily media changes. On *day 3*, cells were rapidly washed three times in ice-cold PBS and 400 µL of cold extraction solution (50% methanol, 30% acetonitrile, and 20% water) was added to each well. After 5 min at 4°C, the extracts were centrifuged (10 min at 16,000 *g*) to remove insoluble material, and the supernatant collected and stored at −80°C until liquid chromatography mass spectrometry (LC-MS) analysis. LC-MS data were normalized to total protein concentrations determined using a modified Lowry assay (15). Wells containing proteins <100 µg were excluded from analysis as several metabolites were not reliably detected in those samples. LC-MS was performed as previously described (16) and relative metabolite levels were normalized to the well protein content. Metabolites were measured in triplicate wells (technical replicates) and the mean of the triplicates considered to be an independent replicate (i.e., *n* = 1). Each experiment was repeated in five independent replicates (i.e., cell passages) and therefore the total *n* = 5.

### Statistical Analysis

Data are presented in bar graphs or line graphs showing mean with SEM. Sample sizes are indicated in the figure legends and data points represent means obtained from independent experiments. Statistical analyses were performed using GraphPad Prism9 using two-tailed Student’s *t* tests, two-way ANOVA with Bonferroni’s multiple comparisons test, or nonlinear regression analyses. *P* values or adjusted P values <0.05 were considered significant.

## RESULTS

### Physiological Medium (Plasmax) Increased hTSC Proliferation and Differentiation

**Figure 1.**
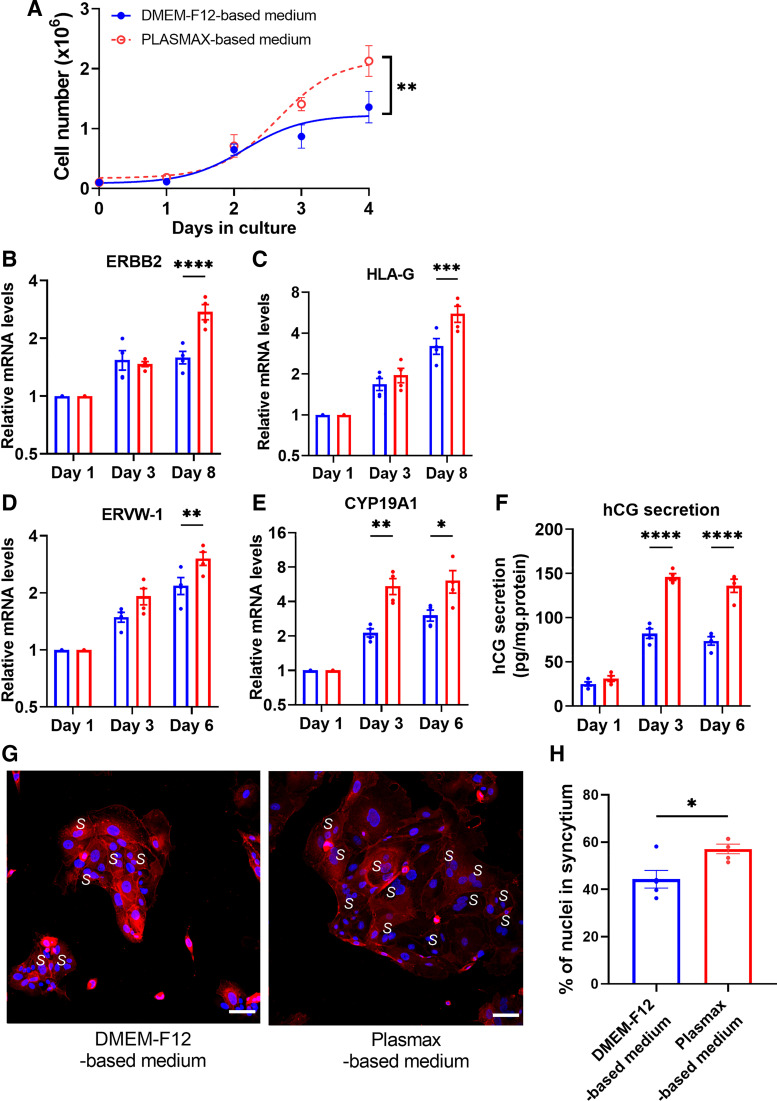
hTSC proliferation and differentiation into STB and EVTs in Plasmax- or DMEM-F12- based medium. *A*: hTSCs were plated in DMEM-F12- or Plasmax-based medium at 0.1 × 10^6^ cells per well and cells were counted after indicated days in culture. hTSCs were differentiated into extravillous trophoblasts (EVTs) or syncytiotrophoblast (STB) and mRNA levels of *ERBB2* (*B*) and *HLA-G* (*C*) were measured in EVT-differentiated cells, and *ERVW-1* (*D*) and *CYP19A1* (*E*) were measured in STB-differentiated cells at the indicated timepoints. *F*: hCG secretion in the media was measured at indicated timepoints following STB differentiation. Syncytial fusion was analyzed by quantifying the distribution of nuclei (blue) within E-cadherin-labeled cellular boundaries (red) following STB differentiation. *G*: representative immunofluorescence images of cells showing nuclei localized in syncytia (*S*). *H*: quantification of the percentage of nuclei in syncytia. Symbols (*A*) and bar graphs (*B–H*) represent means ± SE, *n* = 4 or 5. *P < 0.05, **P < 0.01, ***P < 0.001, ****P < 0.0001. EVT, extravillous trophoblast; hCG, human chorionic gonadotrophin; hTSC, human trophoblast stem cell; STB, syncytiotrophoblast.

Initial proliferation rates of hTSCs were similar in the different media, but hTSCs cultured in Plasmax-based medium showed faster proliferation by *days 3* and *4*, as demonstrated by increased cell numbers (Fig. 1*A*).

hTSCs can be induced to differentiate into STB or EVTs (2). Therefore, we determined if Plasmax-based medium significantly favors trophoblast differentiation compared with DMEM-F12-based medium. hTSCs grown under maintenance conditions were trypsinized and replated as described in the methods using Plasmax- or DMEM-F12-based medium. The mRNA levels of differentiation markers were assessed after 1, 3, and 8 days for EVTs or 1, 3, and 6 days for STB. Cells cultured in Plasmax-based medium show increased EVT markers *ERBB2* (Fig. 1*B*) and *HLA-G* (Fig. 1*C*) after 8 days compared with DMEM-F12-based medium. For cells differentiating along the STB pathway, the STB marker *ERVW-1* was increased after 6 days (Fig. 1*D*) and *CYP19A1* after 3 and 6 days (Fig. 1*E*) in Plasmax-based medium.

To further demonstrate the impact of the different media on function, we measured hCG secretion (as a biochemical marker) and syncytial fusion (as a morphological marker) during STB differentiation. hCG secretion was increased by ∼1.8-fold in Plasmax-based medium after 3 and 6 days of STB differentiation (Fig. 1*F*). Moreover, syncytial fusion as determined by the percentage of nuclei within E-cadherin-stained cell boundaries was increased by ∼1.3-fold in Plasmax-based medium (Fig. 1, *G* and *H*).

### Plasmax Increased hTSC Mitochondrial Function

**Figure 2.**
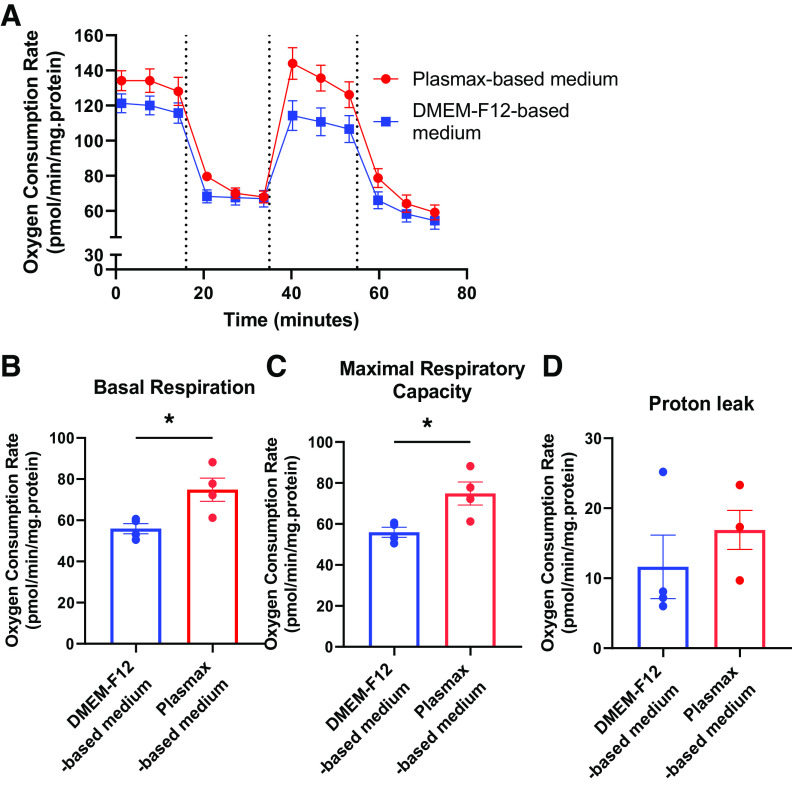
Mitochondrial respiration of hTSCs cultured in Plasmax- vs. DMEM-F12-based medium. *A*: oxygen consumption rate (OCR) was measured via Seahorse bioanalyzer after 3 days of culture in Plasmax- or DMEM/F12-based medium. *B*: basal respiration was calculated by subtracting nonmitochondrial respiration (Rot/AA) from basal OCR. *C*: maximal respiratory capacity is derived by subtracting nonmitochondrial respiration from FCCP rate. *D*: proton leak is assessed by subtracting nonmitochondrial respiration from ATP-linked respiration (oligomycin). Bar graphs represent means ± SE, *n* = 4. *P < 0.05. AA, antimycin a; FCCP, trifluoromethoxy carbonylcyanide phenylhydrazone; hTSC, human trophoblast stem cell; Oligo, oligomycin; Rot, rotenone.

To determine if the alterations in proliferation were associated with changes in mitochondrial function, mitochondrial respiration was assessed using the Seahorse bioanalyzer. hTSCs cultured in Plasmax-based medium for 3 days showed a modestly increased oxygen consumption rate compared with DMEM-F12 (Fig. 2*A*). hTSCs in Plasmax-based medium exhibited ∼30% increase in both basal and maximal respiration, but no changes in proton leak (Fig. 2, *B*–*D*).

### Metabolic Differences between hTSCs Cultured in Plasmax- versus DMEM-F12-Based Medium

**Figure 3.**
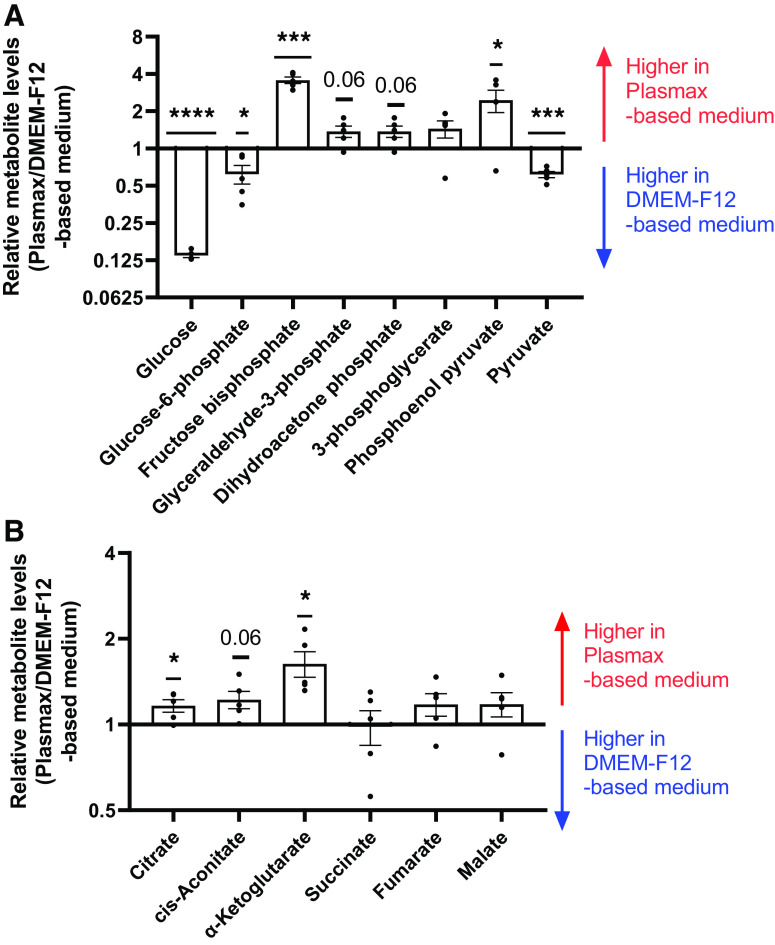
Glycolytic and TCA cycle intermediates are increased with Plasmax-based medium. Glycolytic (*A*) and TCA cycle intermediates (*B*) were measured by LC-MS and expressed as Plasmax/DMEM-F12-based medium. Bar graphs represent means ± SE, *n* = 5. *P < 0.05, ***P < 0.001, ****P < 0.0001. TCA, tricarboxylic.

We next assessed changes in cellular energy metabolism by measuring intracellular levels of glycolytic and tricarboxylic acid (TCA) cycle metabolites by LC-MS. hTSCs in DMEM-F12-based medium had higher levels of glucose and glucose-6-phosphate (Fig. 3*A*), likely due to approximately threefold higher concentrations of glucose in DMEM-F12 compared with Plasmax. However, the downstream glycolytic intermediates fructose-bisphosphate, glyceraldehyde-3-phosphate, and phosphoenolpyruvate were significantly higher in Plasmax-based medium-cultured hTSCs (Fig. 3*A*). Intracellular levels of pyruvate were higher for hTSCs in DMEM-F12-based medium, in-line with the fivefold higher concentrations of pyruvate in DMEM-F12 compared with Plasmax.

The TCA cycle Intermediates citrate, cis-aconitate, and α-ketoglutarate were higher in Plasmax-based medium-cultured hTSCs but the downstream metabolites succinate and malate were not different between the two media (Fig. 3*B*). These differences may be explained by the presence of citrate in Plasmax but not in DMEM-F12.

### Metabolic Indicators of Cellular Redox State and Methylation Capacity in hTSCs Cultured in Plasmax- versus DMEM-F12-Based Medium

**Figure 4.**
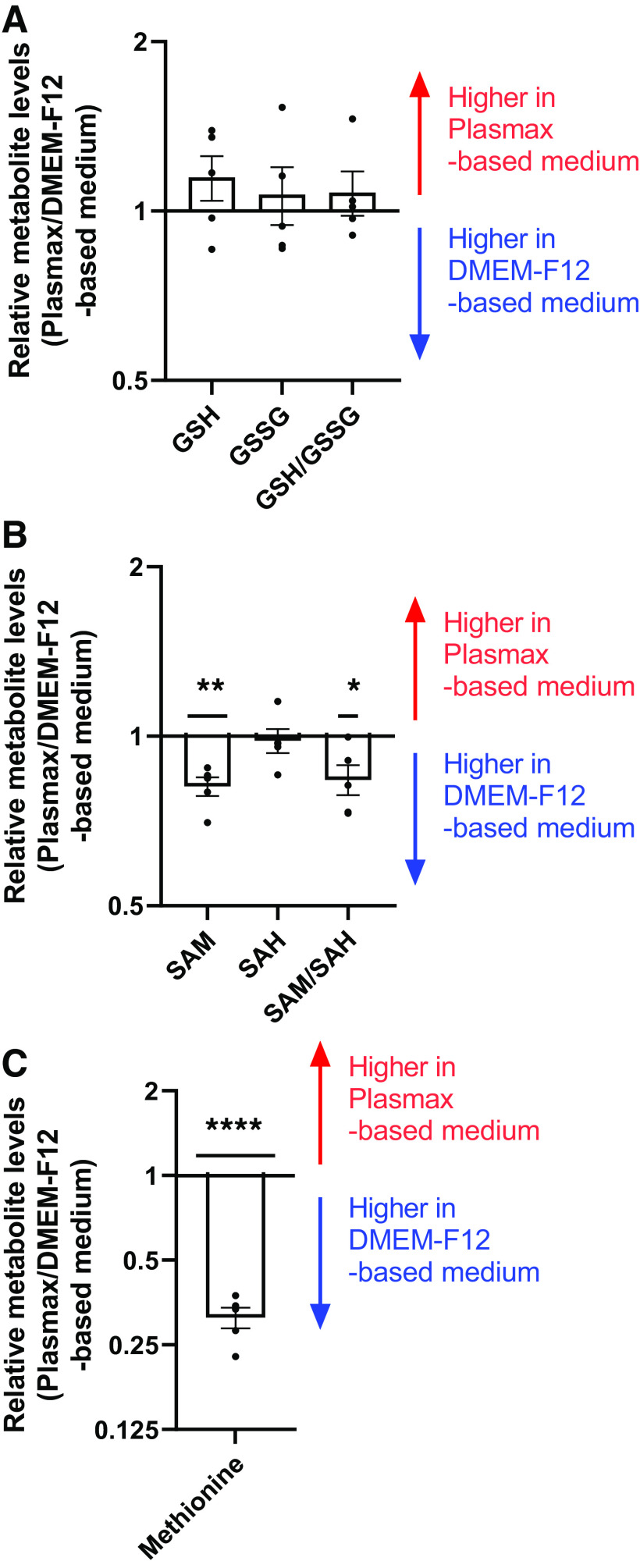
Metabolic indicators of redox state and methylation capacity. GSH, GSSG (*A*), S-adenosylmethionine (SAM), S-adenosyl-homocysteine (SAH) (*B*), and methionine (*C*) were measured by LC-MS and expressed as Plasmax/DMEM-F12-based medium. Bar graphs represent means ± SE, *n* = 5. *P < 0.05, **P < 0.01, ****P < 0.0001. GSH, GSSG, glutathione/glutathione disulfide.

Given the modest increase in mitochondrial respiration in hTSCs cultured in Plasmax-based medium, we sought to determine whether this was associated with an altered redox status. The glutathione/glutathione disulfide (GSH/GSSG) ratio, which is commonly used as an indicator of cellular redox status, was not altered in hTSCs cultured in the different media (Fig. 4*A*).

Lastly, we determined if changes in hTSC differentiation capacity may be due to shifts in metabolic pathways regulating DNA methylation. S-adenosylmethionine (SAM) is a methyl donor in methylation reactions, whereas S-adenosyl-homocysteine (SAH) is a product of these reactions. Thus, SAM/SAH ratio can reflect the methylation capacity of cells. Intracellular levels of SAM, and consequently, SAM/SAH ratio was significantly higher in hTSCs cultured in DMEM-F12-based medium (Fig. 4*B*). This may be explained by the elevated intracellular levels of methionine in hTSCs cultured with DMEM-F12-based medium, as DMEM-F12 contains higher concentrations of this amino acid than Plasmax and human plasma (Fig. 4*C*).

## DISCUSSION

The recent introduction of physiological media (Plasmax and HPLM) is an important development in cell culture studies. However, most of the studies employing these media have been limited to cancer cells, which are highly proliferative, and their metabolism is extensively reprogrammed compared with normal cells. Thus, cancer cell culture in physiological medium results in vast alterations in cell metabolism and behavior (9–11, 17). Despite these recent developments, to date, only one study has compared the effects of physiological versus standard media in noncancerous cells (12).

Plasmax is a chemically defined medium formulated using nutrients, metabolites at concentrations reported for plasma from healthy adult humans (9). Conversely, of the 51 compounds present in DMEM-F12 (Table S1 of Ref. 18) more than half (27/51) are present at greater than twofold higher or lower concentrations compared with the physiological concentrations reported in human plasma (9). Although the concentrations used to develop Plasmax were based on nonpregnant individuals, they are still physiologically relevant for understanding early placental development as major maternal metabolic and physiological adaptations are yet to occur in the first trimester of pregnancy. It is important to note that FBS, a common additive to cell culture media, also contains undefined concentrations of nutrients and other organic compounds. Although most trophoblast culture studies typically use 5–20% FBS to supplement their culture media (8), FBS is used at only 0.2% for hTSCs therefore the nutrient composition of the basal medium employed is crucial to support their metabolic needs.

hTSCs cultured in Plasmax-based medium showed higher proliferation and differentiation, despite lower concentrations of many nutrients in Plasmax-based compared with DMEM-F12-based medium, including glucose. The specific component that mediates these differences was not examined in this study as it would require systematic investigation of every compound that is present at different concentrations in the two media. However, it is possible that the supraphysiological levels of certain metabolites present in DMEM-F12 may attenuate cell proliferation. For example, high concentrations of pyruvate in DMEM-F12 were previously shown to induce a state of pseudohypoxia in cancer cells (9).

The increased proliferation of hTSCs cultured in Plasmax-based medium was consistent with alterations in hTSC metabolism. First, Plasmax-cultured hTSCs demonstrated increased levels of glycolytic intermediates, despite lower levels of glucose in Plasmax-based medium suggesting a metabolic reprogramming of central carbon metabolism. Second, several TCA intermediates were increased with Plasmax-based medium. And lastly, mitochondrial respiration was elevated in hTSCs cultured in Plasmax-based medium. Although increases in mitochondrial respiration can sometimes lead to elevated mitochondrial ROS, the redox state of the cells (as determined by GSH/GSSG ratio) was not different. These findings suggest that the culture media-dependent alterations in the metabolic state of hTSCs were not associated with oxidative stress.

Previous studies in term trophoblasts have shown that the progenitor cytotrophoblast cells maintain higher levels of mitochondrial respiration compared with differentiated STB (19, 20). We did not examine the effects of the different media on mitochondrial respiration in the differentiated trophoblasts (either STB or EVTs) and therefore we are unable to conclude whether the effects on mitochondrial function are maintained following differentiation.

One of the defining features of hTSCs is their ability to differentiate into STB and EVTs (21). Differentiation into both these lineages was significantly increased in hTSCs cultured in Plasmax-based medium. The role of metabolism in regulating trophoblast differentiation is largely unknown. The placenta is globally hypomethylated compared with other somatic tissues (22) and this is believed to be necessary for the transcription of placental-specific genes such as *ERVW-1*, which are involved in differentiation (23, 24). SAM is a product of the methionine cycle and a methyl donor for DNA methylation reactions. SAH on the other hand is both a product and inhibitor of methylation reactions. Thus, the lower SAM/SAH ratio in hTSCs cultured in Plasmax-based medium may reflect a state of hypomethylation increasing differentiation capacity.

In conclusion, hTSCs cultured in the physiological Plasmax-based medium show improved proliferation and differentiation capacity that was associated with alterations in their metabolism. Thus, the use of physiological media may be important in understanding the mechanisms that underlie these processes.

## DATA AVAILABILITY

Data will be made available upon reasonable request.

## GRANTS

This work was funded by the Cancer Research UK award (A23982) to S. Tardito, and by the MRC Career Development Award (MR/W027046/1) to I. L.M.H. Aye, and a PhD scholarship from the Centre for Trophoblast Research, University of Cambridge to G. Avellino.

## DISCLOSURES

No conflicts of interest, financial or otherwise, are declared by the authors.

## AUTHOR CONTRIBUTIONS

I.L.M.H.A. conceived and designed research; G.A., R.D., S.N.R., and I.L.M.H.A. performed experiments; G.A., R.D., S.N.R., S.T., and I.L.M.H.A. analyzed data; G.A., S.N.R., D.S.C-J., G.C.C.S., S.T., and I.L.M.H.A. interpreted results of experiments; G.A. and I.L.M.H.A. prepared figures; G.A. and I.L.M.H.A. drafted manuscript; G.A., D.S.C-J., G.C.S.S., S.T., and I.L.M.H.A. edited and revised manuscript; G.A., R.D., S.N.R., D.S.C-J., G.C.S.S., S.T., and I.L.M.H.A. approved final version of manuscript.
